# Dual-response detection of Ni^2+^ and Cu^2+^ ions by a pyrazolopyrimidine-based fluorescent sensor and the application of this sensor in bioimaging†

**DOI:** 10.1039/c9ra06227k

**Published:** 2019-11-04

**Authors:** Yun-Qiong Gu, Wen-Ying Shen, Yan Mi, Yan-Fang Jing, Jing-Mei Yuan, Peng Yu, Xiao-Min Zhu, Fei-Long Hu

**Affiliations:** Guangxi Key Laboratory of Chemistry and Engineering of Forest Products, Guangxi University for Nationalities Nanning 530006 China; School of Environment and Life Science, Nanning Normal University Nanning 530001 Guangxi China; Key Laboratory for the Chemistry and Molecular Engineering of Medicinal Resources (Guangxi Normal University), Ministry of Education of China China

## Abstract

Herein, a dual-response fluorescent sensor, L, based on pyrazolopyrimidine was designed and developed for the simultaneous detection of Ni^2+^ and Cu^2+^ ions in the presence of other metal ions; the structural characterization of L was carried out by FTIR spectroscopy, NMR spectroscopy, HRMS and X-ray diffraction analysis. The sensor L effectively displayed fluorescence quenching towards the Ni^2+^ and Cu^2+^ ions with high sensitivity without interference from other metal ions. The results reveal that L binds to Ni^2+^ and Cu^2+^ in a 2 : 1 pattern, which matches well with the result of the Job's plot. The association constants of L with Ni^2+^ and Cu^2+^ were 3.2 × 10^4^ M^−1^ and 7.57 × 10^4^ M^−1^, respectively. The detection limits (DLs) are down to 8.9 nM for Ni^2+^ and 8.7 nM for Cu^2+^. The fluorescence imaging of L in T-24 cells was investigated because of the low cytotoxicity of L, indicating that L could be used to detect Ni^2+^ and Cu^2+^ in living cells.

## Abstract 2

A pyrazolopyrimidine-based fluorescent sensor L was developed and applied for detection of Cu^2+^ and Ni^2+^ in ethanol solution by photoluminescence quenching. It shows low cytotoxicity and good imaging characteristics for Cu^2+^ and Ni^2+^ in living cells.

## Graphical abstract

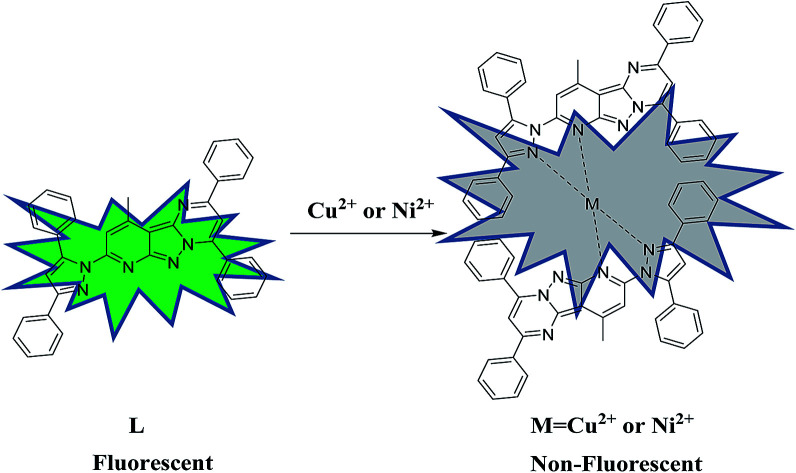

## Introduction

Heavy metal ions have gained extensive attention due to their importance in many biological and environmental processes. However, the ingestion of these metals, including arsenic, mercury, copper, nickel, and lead,^1^ even at very low concentrations can lead to numerous health issues or toxic effects.^2^ Copper is associated with various biological processes^3^ as it is utilized in important cofactors of several proteins and enzymes.^4–7^ The imbalance of Cu^2+^ level causes various diseases; moreover, nickel at high concentrations can cause severe harmful effects such as pneumonitis, lung cancer, respiratory problems, and kidney damage through its bioaccumulation.^8–10^ If the accumulation of excess amounts of copper and nickel ions or their misregulation in the body, it is very dangerous to man and animals.^11,12^ Hence, the sensing of copper and nickel ions in aqueous media is necessary because of the vital roles of these ions in biological systems.

Nowadays, spectroscopic techniques, such as atomic absorption spectroscopy (AAS), ion-chromatography ultraviolet-visible spectroscopy (IC-UV-vis), inductively coupled plasma mass spectroscopy (ICP-MS), and chromatography, are being used to detect the Cu^2+^ and Ni^2+^ metal ions.^13–17^ These traditional methods have high sensitivity;^18^ however, they require expensive instruments, trained professionals, difficult sample preparation processes and high time consumption;^19^ therefore, continuous efforts have been focused on the development of methods for the rapid and convenient sensing of particular heavy metal cations.^20,21^ Although many fluorescent sensors have been reported for the Cu^2+^ and Ni^2+^ ions, there is still an intense demand for new, efficient Cu^2+^ and Ni^2+^ ion fluorescent probes that can work in aqueous media with high selectivity and sensitivity at low concentrations.

Herein, the luminescent pyrazolopyrimidine 2-(3,5-diphenyl-pyrazol-1-yl)-4-methyl-6,8-diphenyl-1,5,8*a*,9-tetraaza-fluorene (L) was studied. It possesses both pyrimidine and pyrazole moieties as backbones with a phenyl substituent, which can be modified to enhance the fluorescence intensity.^22^ Moreover, due to their good binding characteristics and structures that enable easy modification,^23–28^ pyrazolopyrimidine and its derivatives are vital chelators and sensors for metal ions. The ingenious sensor L is an “N–C–N”-type ligand containing two coordination sites, which can provide a special coordination environment that increases the binding efficiency of L with metal ions.^29–32^ In addition, the introduction of a typical electron-withdrawing substituent, phenyl, can effectively improve the selectivity of L towards metal ions.^33^ The most important thing is that the sensor L shows low toxicity in living cells and may have potential therapeutic value in the diagnosis and treatment field. Therefore, herein, a dual function and low cytotoxic sensor for the label-free detection of Cu^2+^ and Ni^2+^ cations with very low detection limit of nanomolar level in the presence of other cations was synthesized, and then, its potential applications in human cancer cell imaging were investigated.

## Experimental

### Materials

All the materials and solvents were applied as received without further purification. The stock solutions of the sensor L (2 × 10^−3^ M) were prepared by dissolving L in ethanol and then diluted to desired concentration with ethanol when needed. The stock solutions of metal ions (2 × 10^−3^ M) were prepared from the salts LiCl, NaCl, KCl, MgCl_2_·6H_2_O, CaCl_2_·2H_2_O, BaCl_2_·2H_2_O, MnCl_2_·4H_2_O, FeCl_2_·4H_2_O, CoCl_2_·6H_2_O, NiCl_2_·6H_2_O, CuCl_2_·2H_2_O, ZnCl_2_·6H_2_O, CdCl_2_·H_2_O, HgCl_2_, AlCl_3_·6H_2_O, FeCl_3_·6H_2_O, AgNO_3_ and Pb(NO_3_)_4_ using pure water.

### Instruments

^1^H NMR spectra were obtained by the Bruker Avance 400 MHz spectrometer in *d*_6_-DMSO, and high-resolution mass (HRMS) spectra were obtained using the Agilent 6224 TOF LC/MS instrument. Fourier transform infrared (FTIR) spectroscopy was conducted using the NEXUS 870 FTIR spectrophotometer, and fluorescence spectra were obtained using the Hitachi F-4600 spectrofluorometer. Quantum yields were determined with respect to quinine sulfate (*Φ*_F_ = 0.546) using the standard method;^34–36^ the X-ray crystallography data and the results of elemental analyses were obtained using the Bruker Apex II X-ray diffractometer and the PerkinElmer Series II CHNS/O 2400 analyzer, respectively. The images of living cells were obtained using the Cyration 5 confocal microscope.

### Synthesis

#### 2-(3,5-Diphenyl-pyrazol-1-yl)-4-methyl-6,8-diphenyl-1,5,8*a*,9-tetraaza-fluorene (L)

6-Hydrazinyl-4-methyl-2*H*-pyrazolo[3,4-*b*]pyridine-3-amine (A) was prepared according to a method reported in the literature.^37^

1,3-Diphenylpropane-1,3-dione (0.448 g, 2.0 mmol) was dissolved in ethanol (20 mL), and then, an ethanol solution (10 mL) of A (0.353 g, 1.0 mmol) was added to the abovementioned solution. The resulting mixture was heated under reflux for 8 h, and then, the solution was cooled down to room temperature. A bright yellow solid was formed, which was filtered, washed with ice-cold ethanol and dried to obtain the yellow solid L (0.762 g yield: 91.7%). ^1^H NMR (400 MHz, *d*_6_-DMSO, Fig. S1†) *δ* 8.51 (dd, *J* = 8.0, 1.4 Hz, 2H, benzene-H), 8.33 (s, 1H, pyrimidine-H), 8.28–8.22 (m, 2H, benzene-H), 8.01 (d, *J* = 7.1 Hz, 2H, benzene-H), 7.68–7.66 (m, 2H, benzene-H), 7.65 (s, 1H, pyridine-H), 7.64 (s, 2H, benzene-H), 7.62 (s, 2H, benzene-H), 7.50 (t, *J* = 7.5 Hz, 2H, benzene-H), 7.44–7.38 (m, 2H, benzene-H), 7.38–7.31 (m, 4H, benzene-H), 7.28 (s, 1H, pyrazole-H), 3.17 (s, 3H, Py-CH_3_). HRMS: 555.2243 [M + H^+^] (Fig. S2†). Anal. calc. (for C_37_H_26_N_6_) C 80.12; H 4.72; N 15.15%, found. C 80.15; H 4.74; N 15.11%. IR (KBr): 3456, 1631, 1382, 1061, 820 cm^−1^ (Fig. S3†).

#### Cu complex

The Cu complex was prepared by treating L (0.0554 g, 0.1 mmol) with CuCl_2_·6H_2_O (0.0178 g, 0.1 mmol) in C_2_H_5_OH–H_2_O (same as used in detection) (3 : 1 by volume, 32 mL). Microcrystals were obtained after evaporating the blue solution slowly at room temperature, which were filtered off, washed with cold ethanol and dried *in vacuo*. (Yield: 68% base on CuCl_2_·6H_2_O). HRMS: 1171.3727 [Cu(L)_2_]^+^ (calc. 1171.3834) (Fig. S4†).

### Cell culture and fluorescence imaging for Cu^2+^ and Ni^2+^ in living cells

The living cells were obtained from the Shanghai Cell Bank of the Chinese Academy of Science, which were grown in the Dulbecco^'^s modified Eagle medium (DMEM, Gibco), containing 10% fetal bovine serum (FBS, Gibco) (V/V), under an atmosphere of 5% CO_2_ and 95% air at 37 °C. These cells were incubated with the compound L (5 μM) under the same abovementioned experimental conditions for 4 h. The cells were washed with PBS buffer and simultaneously imaged using a confocal laser scanning microscope. Then, the cells were incubated with the CuCl_2_ and NiCl_2_ solutions (30 μM) for another 6 h, washed with PBS buffer and again imaged using a confocal laser scanning microscope.

## Results and discussion

### Synthesis and characterization

**Scheme 1:**
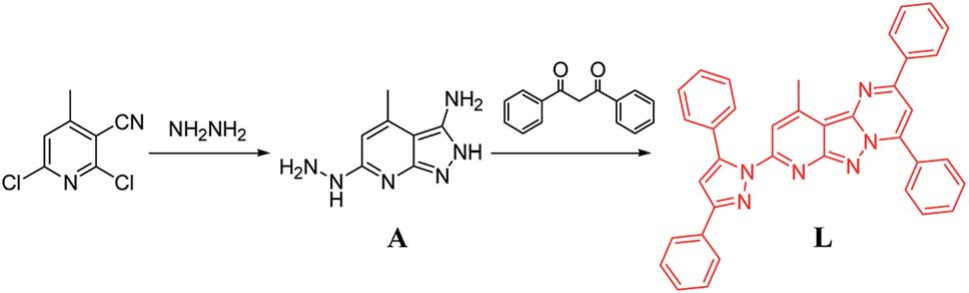
Synthesis of the sensor L.

**Fig. 1:**
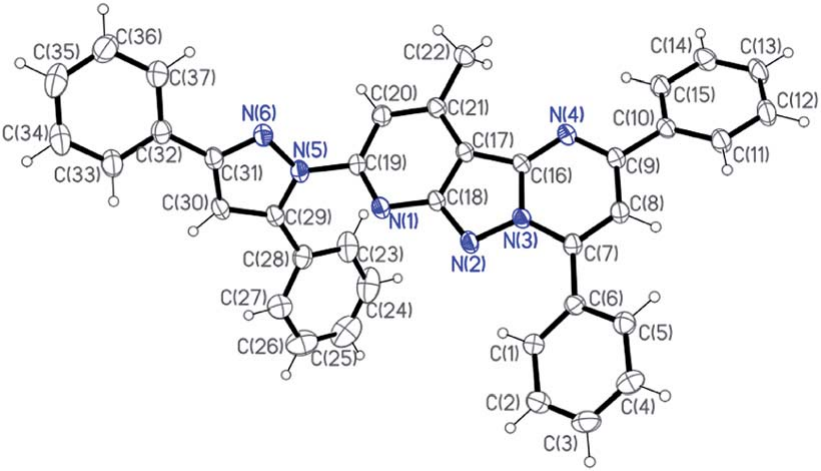
The crystal structure of the chemosensor L.

As illustrated in Scheme 1, the target chemosensor L was easily synthesized by the condensation reaction of pyridyl hydrazine A with 1,3-diphenylpropane-1,3-dione. Subsequently, its structure was characterized by ^1^H NMR spectroscopy, FTIR spectroscopy, HRMS and single-crystal X-ray crystallography; all the spectroscopic data were in agreement with the formula of L. Both pyrazole and pyrimidine units from the same ligand coordinated to the same Ni/Cu atom forming a conjugated plan motif. As shown in Fig. 1, multiple binding sites were provided by the electron-donating N atom; a chelating model was suggested based on the crystal structure when L was treated with the metal ions.

### Metal-ion selectivity

**Fig. 2:**
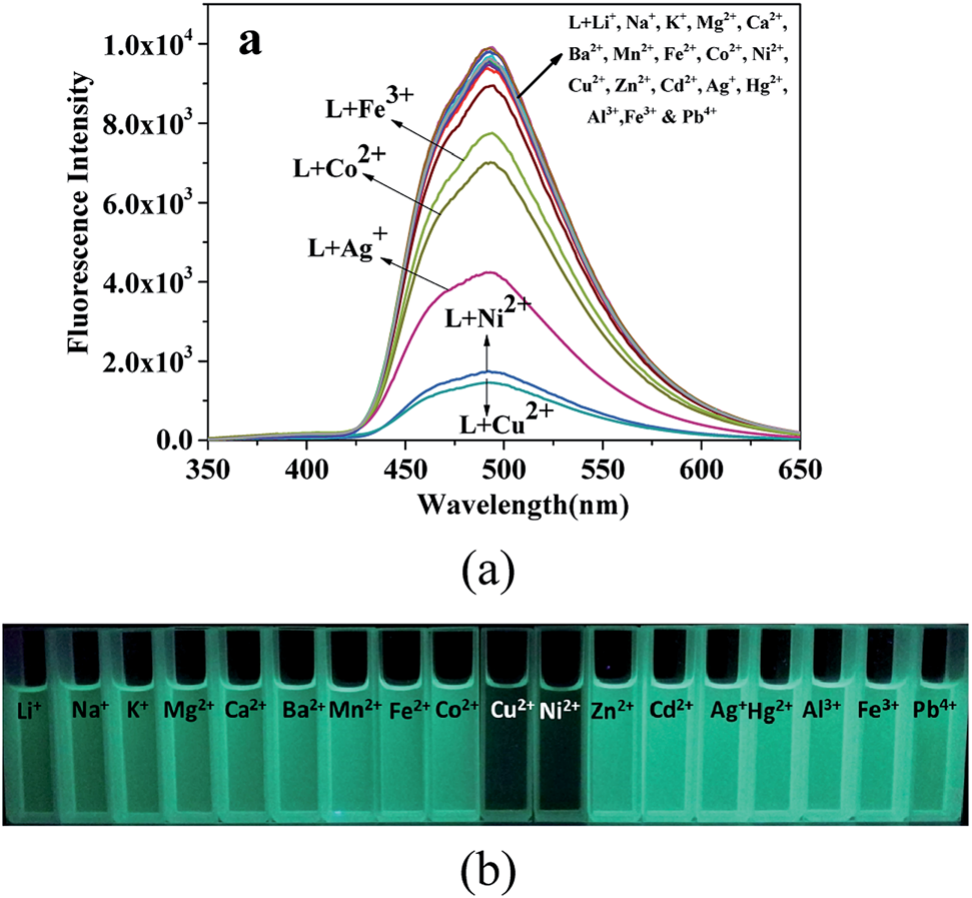
(a) The fluorescence spectra of L (20.0 μM, *λ*_ex_ = 330 nm) in the absence and presence of various metal ions (2.5 equiv.) in an ethanol solution. (b) The colour changes of L in the presence of various metal ions under a 365 nm UV lamp.

The fluorescence sensing selectivity of L (2 × 10^−5^ M in ethanol) was investigated towards common metal ions (2.5 equiv.), such as Li^+^, Na^+^, K^+^, Mg^2+^, Ca^2+^, Ba^2+^, Mn^2+^, Fe^2+^, Co^2+^, Ni^2+^, Cu^2+^, Zn^2+^, Cd^2+^, Hg^2+^, Al^3+^, Fe^3+^, and Pb^4+^, by determining the changes in the fluorescence intensity. The probe L exhibited an intense emission band at 494 nm (*λ*_ex_ = 330 nm) with a high quantum yield determined using quinine sulfate as a reference.^35^ The addition of the Cu^2+^ and Ni^2+^ ions to the ethanol solution of probe L caused a remarkable fluorescence quenching ((*I*_0_ − *I*)/*I*_0_ × 100% = 81.9% for the Ni^2+^ ions and 84.8% for Cu^2+^) at 494 nm, and slightly quenched (addition of Fe^3+^, Co^2+^ or Ag^+^) or no fluorescence spectral changes were observed upon the addition of other metal ions under the same conditions (Fig. 2a). This result clearly suggests that L may be used to detect the Ni^2+^ and Cu^2+^ ions due to their higher binding affinity with the chelator of L that leads to the formation of stable complexes between L and the Ni^2+^ or Cu^2+^ ions.^38–40^ However, other metal ions caused a slight change in the fluorescence intensity due to the unsuitable coordination geometry conformation of the chemosensor L and inappropriate ionic radius and insufficient binding energy of these metal ions.^41^ As shown in Fig. 2b, the fluorescence clearly changed from bright green to colourless under UV light (365 nm) after the addition of the Ni^2+^ and Cu^2+^ ions, whereas no change was observed after the addition of other metal ions tested herein under UV light. These findings revealed that the sensor L could be used to easily detect the Ni^2+^ and Cu^2+^ ions by the naked eye under 365 nm UV light.

### Competitive studies

**Fig. 3:**
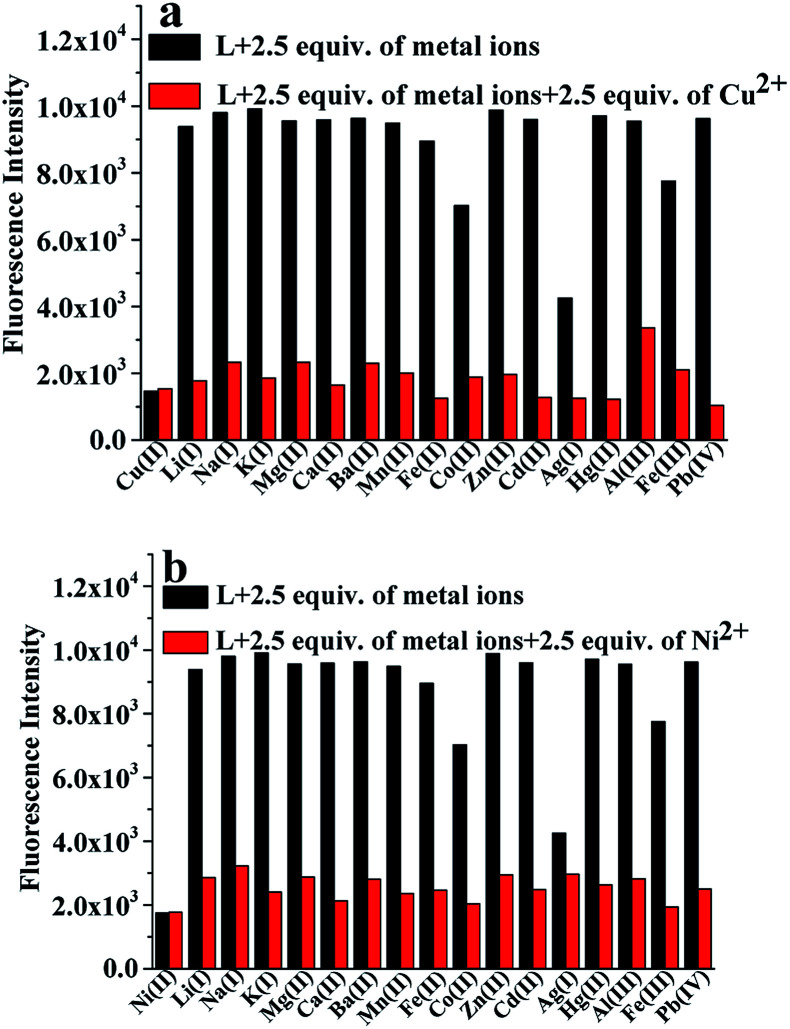
(a) The fluorescence intensity at 494 nm for the sensor L (20.0 μM) with 2.5 equiv. of various metal ions (black bars) in an ethanol solution and that after the addition of 2.5 equiv. Cu^2+^ (red bars) to the abovementioned solution. (b) The fluorescence intensity at 494 nm for the sensor L (20.0 μM) with 2.5 equiv. of various metal ions (black bars) in an ethanol solution and that after the addition of 2.5 equiv. Ni^2+^ (red bars) to the abovementioned solution.

To confirm the selectivity of L, competitive experiments towards Cu^2+^ or Ni^2+^ (2.5 equiv.) over other competitive coexisting metal ions (2.5 equiv.) were carried out. The changes in the fluorescence intensity are displayed in Fig. 3a and b. As shown in Fig. 3, except for Ni^2+^ and Cu^2+^, other competitive metal ions had no obvious effect on the fluorescence of L. This performance clearly indicated that the binding interaction between L and Ni^2+^ or Cu^2+^ was stronger than that between L and other metal ions. Therefore, this shows that the recognition of Ni^2+^ and Cu^2+^ by L is barely interfered by other coexisting metal ions; this indicates that L is a dual-response fluorescent sensor for the detection of Ni^2+^ and Cu^2+^ ions in an ethanol solution.

### Titration analysis

**Fig. 4:**
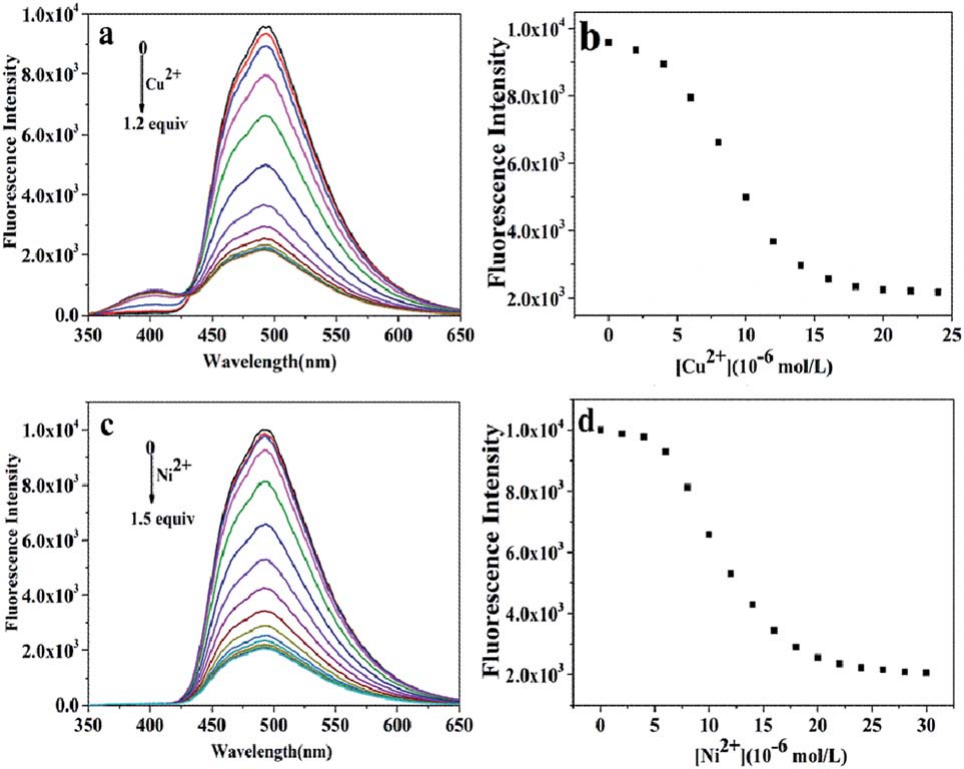
(a) Fluorescence spectra of L (20.0 μM) in the presence of different concentrations of Cu^2+^ in an ethanol solution; (b) the plot of the fluorescence intensity of L as a function of Cu^2+^ concentration; (c) fluorescence spectra of L (20.0 μM) in the presence of different concentrations of Ni^2+^ in an ethanol solution; and (d) the plot of the fluorescence intensity of L as a function of Ni^2+^ concentration.

**Fig. 5:**
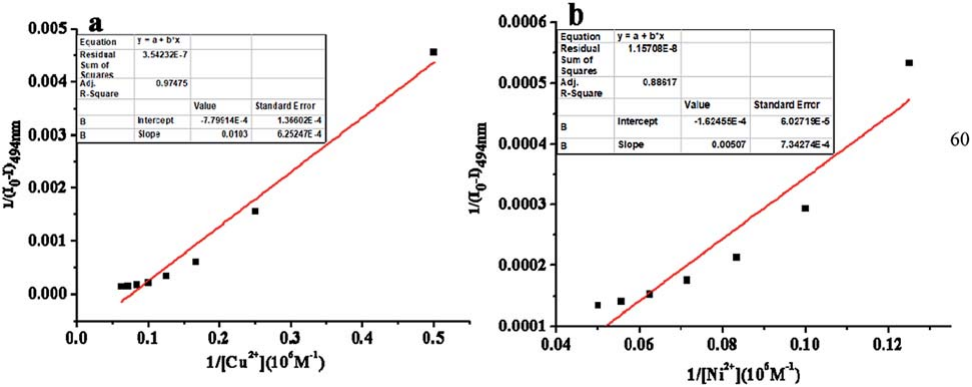
Benesi–Hildebrand plot of the Cu complex of L (a) and Ni complex of L (b) in an ethanol solution.

To obtain a better insight into the sensitivity of the probe L, we increased the concentration of Cu^2+^ (0–1.2 equiv.) or Ni^2+^ (0–1.5 equiv.) ions to detect the fluorescence intensity of L (20.0 μM). The strong fluorescence intensity of the sensor L at 494 nm (quantum yield, *Φ*_F_ = 0.293) was attributed to the fluorescence enhancement effect of the N dopant atoms on the surface of L in the excited state.^34^ The changes in the fluorescence intensity as a function of the concentration of Cu^2+^ and Ni^2+^ ions are shown in Fig. 4a and c, respectively. The fluorescence intensity gradually decreased and then reached minimum with an increase in the concentration of the Cu^2+^ and Ni^2+^ ions (Fig. 4b and d), respectively. Based on these concentration-dependent experiments, the corresponding association constants of L with Ni^2+^ and Cu^2+^ were calculated to be 3.2 × 10^4^ M^−1^ and 7.57 × 10^4^ M^−1^ by the Benesi–Hildebrand plot, with the correlation coefficient *R* = 88.617 and 97.475, respectively (Fig. 5). A linear regression curve was fitted to the titration data of the fluorescence intensity and the concentration of Ni^2+^ and Cu^2+^, and then, the detection limit (DL) values were calculated to be 8.9 nM and 8.7 nM (Fig. S5†), respectively, which were determined reasonably by the equation DL = *K* × *σ*/*S* (eqn (S1)†), where *K* = 3, *σ* is the standard deviation of the blank solution (10 times) and *S* is the slope of the calibration curve;^42^ moreover, these detection limits were significantly less than the maximum contaminant levels of Ni^2+^ (17 μM) and Cu^2+^ (30 μM) in potable water according to the EPA guidelines;^43^ this indicated that the as-prepared L could be deemed as an excellent fluorescent sensor and employed for the recognition and detection of the Ni^2+^ and Cu^2+^ ions instantly in biological and environmental systems.

### Binding mode studies

**Fig. 6:**
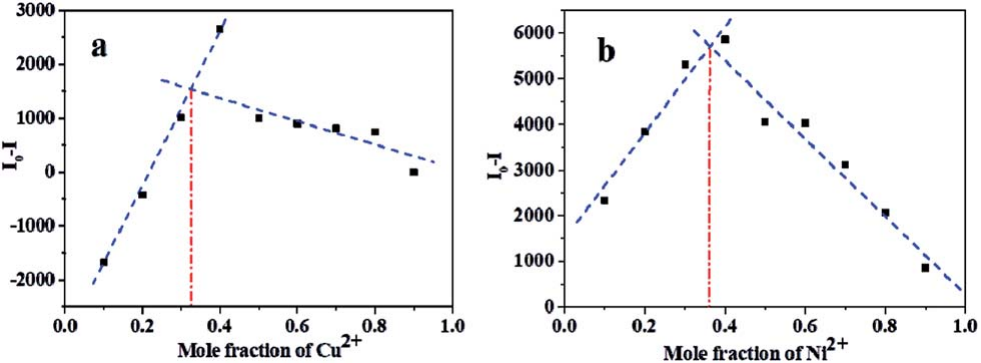
Job's plot for the Cu complex of L (a) and Ni complex of L (b) in ethanol solution.

To obtain further information about the binding mechanism, the binding process was studied by Job's plot and HRMS mass spectral analysis. The Job's plot for L with the metal ions was obtained for achieving the binding stoichiometry between L and Ni^2+^ and Cu^2+^ (Fig. 6). It exhibited a maximum point at a mole ratio fraction of 1/3, indicating a 2 : 1 stoichiometry of the binding mode of L with Ni^2+^ or Cu^2+^.^44^ The HRMS spectra illustrated that the binding ratio of L to Cu^2+^ was 2 : 1 (Fig. S4†). Therefore, the results obtained from the mass spectral analysis correlate well with those obtained by the Job's method.

### Fluorescence imaging in T-24 cells

Cytotoxicity tests were carried out to ascertain the potential biological applications of L*in vitro*. The cytotoxicity of L against seven human tumor cell lines (T-24, HeLa, Hep-G2, MGC803, A549, NCI-H460, and SK-OV-3) and a normal liver cell line (HL-7702) was investigated by the MTT method. We treated different cell lines with different concentrations of L for 48 h, and the IC_50_ values ranged from 30 to 40 μM (Table S2†). Overall, the IC_50_ value of L was 39.94 ± 0.28 μM against T-24, and it showed the lowest toxic effect on the T-24 cells, which indicated the good biocompatibility of L in the cells.^28^

**Fig. 7:**
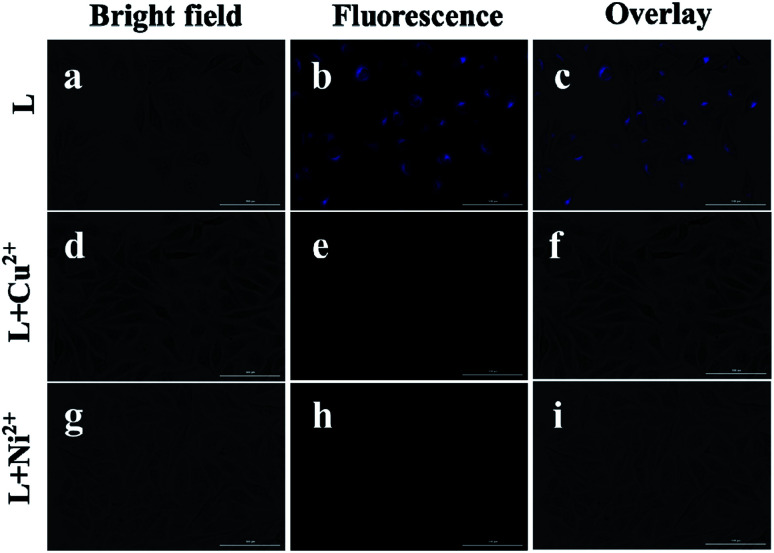
Fluorescence images of the living T-24 cells incubated with L (5 μM) for 4 h (a–c), then treated with Cu^2+^ (30 μM) for another 6 h (d–f) and treated with Ni^2+^ (30 μM) for another 6 h (g–i). (c, f and i) overlay images of (a, b), (d, e) and (g, h); (a, d and g) bright field images of the cell culture.

Inspired by the low cytotoxicity and the strong fluorescence intensity of L, fluorescence imaging to recognize the Ni^2+^ and Cu^2+^ ions in living cells was conducted (Fig. 7). The fluorescence filter of the blue channel with an excitation of 330 nm was chosen as the signal output. The T-24 cells were first treated with 5 μM of L for 4 h at 37 °C and then washed with PBS buffer, which displayed a blue fluorescence (Fig. 7b and c). Consequently, it was observed that the fluorescence was quenched after the incubation of cells with 30 μM of a CuCl_2_·6H_2_O or NiCl_2_·6H_2_O solution for another 6 h (Fig. 7e and h). These imaging experiments prove good permeability of L into the membrane of cells, and thus, L can be used to recognize these two metal ions in living cells.

## Conclusions

In conclusion, herein, an easy-to-prepare and efficient dual-response fluorescent sensor, L, for the detection of Ni^2+^ and Cu^2+^ in an ethanol solution was designed and synthesized, which was investigated in detail by employing fluorescence measurements. The presented sensor L exhibited good sensitivity and biocompatibility for the detection of Ni^2+^ and Cu^2+^ ions over several metal ions with the detection limit of as low as 8.9 nM for Ni^2+^ and 8.7 nM for Cu^2+^. The 2 : 1 binding mode between L and the Ni^2+^ or Cu^2+^ ions was suggested *via* the Job's method and HRMS spectra. The probe L could be used as a staining reagent in cells due to its good biocompatibility, which provided a sensitive response to the fluorescence of Ni^2+^ and Cu^2+^ in living cells. Therefore, it can be a good candidate to recognize Ni^2+^ and Cu^2+^ in a biological environment.

## Conflicts of interest

There are no conflicts to declare.

## Supplementary Material

RA-009-C9RA06227K-s001

RA-009-C9RA06227K-s002
