# Supplementary material for: Dual-response detection of Ni2+ and Cu2+ ions by a pyrazolopyrimidine-based fluorescent sensor and the application of this sensor in bioimaging
Source: RSC Adv. 2019 Nov 4;9(61):35671–6. doi: 10.1039/c9ra06227k (PMC9074711; doi:10.1039/c9ra06227k)
Supplement: RA-009-C9RA06227K-s001 [file RA-009-C9RA06227K-s001.pdf]

Electronic Supplementary Material (ESI) for RSC Advances.
This journal is © The Royal Society of Chemistry 2019

Electronic Supplementary Information (ESI)

Dual-response detection of Ni^{2+} and Cu^{2+} ions by pyrazolopyrimidine-based fluorescent sensor and its application in bioimaging

Yun-Qiong Gu[#b,c], Wen-Ying Shen[#c], Yan Mi[a], Yan-Fang Jing[a], Jing-Mei Yuan[c], Peng Yu[b], Xiao-Min Zhu[c], Fei-Long Hu[*a]

a Guangxi Key Laboratory of Chemistry and Engineering of Forest Products, Guangxi University for Nationalities, Nanning, 530006, China.

b School of Environment and Life Science,Nanning Normal University, Guangxi, Nanning, 530001, China.

c Key Laboratory for the Chemistry and Molecular Engineering of Medicinal Resources (Guangxi Normal University), Ministry of Education of China

Correspondence and requests for materials should be addressed to F.L.H. (*hflphd@163.com*)

Table of Contents

Experimental section...S3

Fig. S1. The ^{1}H NMR (400 M, d_6-DMSO) spectra of **L**..S3

Fig. S2. HRMS spectrum of ligand **L**..S4

Fig.S3. FT-IR spectra of **L**..S5

Table S1. Crystallographic data and refinements of **L**....... ....S5

Fig. S4. HRMS spectrum of complex **L-Cu^{2+}**..S7

Fig. S5. Calculation of detection limits of L for Cu^{2+} (a) and Ni^{2+} (b) in EtOH solution. The spectra were recorded with excitation wavelength at 330 nm and emission wavelength at 494 nm. Fluorescence intensity is the sensor L solution (20 μM) upon addition of different amounts of Cu^{2+}/ Ni^{2+}, respectively.. ....S8

Table S2. Cytotoxicity of the tested compound **L** against different cell lines...............................S8

1 Experimental section

1.1 Single crystal X-ray diffraction analysis

Single crystal X-ray data for **L** was collected on a Bruker Apex II X-ray diffractomete equipped with Mo Kα radiation (λ=0.71073 Å) at room temperature. The integrated intensity data for each reflection was collected by reduction of the data frames with the program Apex II. Hydrogen atoms were located in perfect positions and set riding on individual parent atoms. The structures were solved with the program *SHELXS-2014* and refined by full-matrix least squares on F^2 using the program *SHELXTL-2014*. All non-hydrogen atoms were refined with anisotropic thermal parameters. The parameters used intensity collection and refinements of **L** are summarized in Table S1. The CCDC code for **L** is 1936105.

1.2 Job plot measurements

L (11.08 mg, 2×10^{-3} M) was dissolved in ethanol (10 mL) and then 100, 90, 80, 70, 60, 50, 40, 30, 20, 10 or 0 μL aliquot of this solution was transferred to a vial and diluted with 2.9 mL of ethanol. The same concentration of $CuCl_2$ and $NiCl_2$ solutions was prepared with deionized water and a 0, 10, 20, 30, 40, 50, 60, 70, 80, 90 or 100 μL aliquot of $CuCl_2$ or $NiCl_2$ solutions was added to each diluted **L** solution. Each vial had a total volume of 3 mL. After stirring for one minute, the fluorescence spectrum was collected at room temperature. Job's plots were drawn by plotting ΔI *vs* the mole fraction of metal ions, where ΔI= change of fluorescence intensity at 494 nm.

1.3 Cytotoxicity Assay

The cancer cells were seeded in 96-well culture plates ([(5×10^3)/180 μL]/ well). Cells were cultured for 24 h to reach 70% confluence before treatment, and an amount of 20 μL of tested various concentrations of compound **L** was added to each well. The final concentrations of the tested compound were 5 μM, 10 μM, 20 μM, 50 μM and 100 μM, respectively. The microtiter plates were incubated in humidified atmosphere of 5% CO_2 and 95% air at 37 ^{o}C for another 48 h. Then freshly prepared 3-(4,5-

dimethylthiazol-2-yl)-2,5-diphenyltetrazolium bromide (MTT) solution (10 μL, 5 mg/mL) was added to each well for 4 h. Then the supernatant was removed, and DMSO (100 μL) was added to dissolve the formazan crystals. The absorbance was read on a microplate reader at 490/630 nm. The cytotoxicity was assessed based on the percentage of cell survival compared with the negative control. The IC_{50} values were calculated by the Bliss method (n = 5).

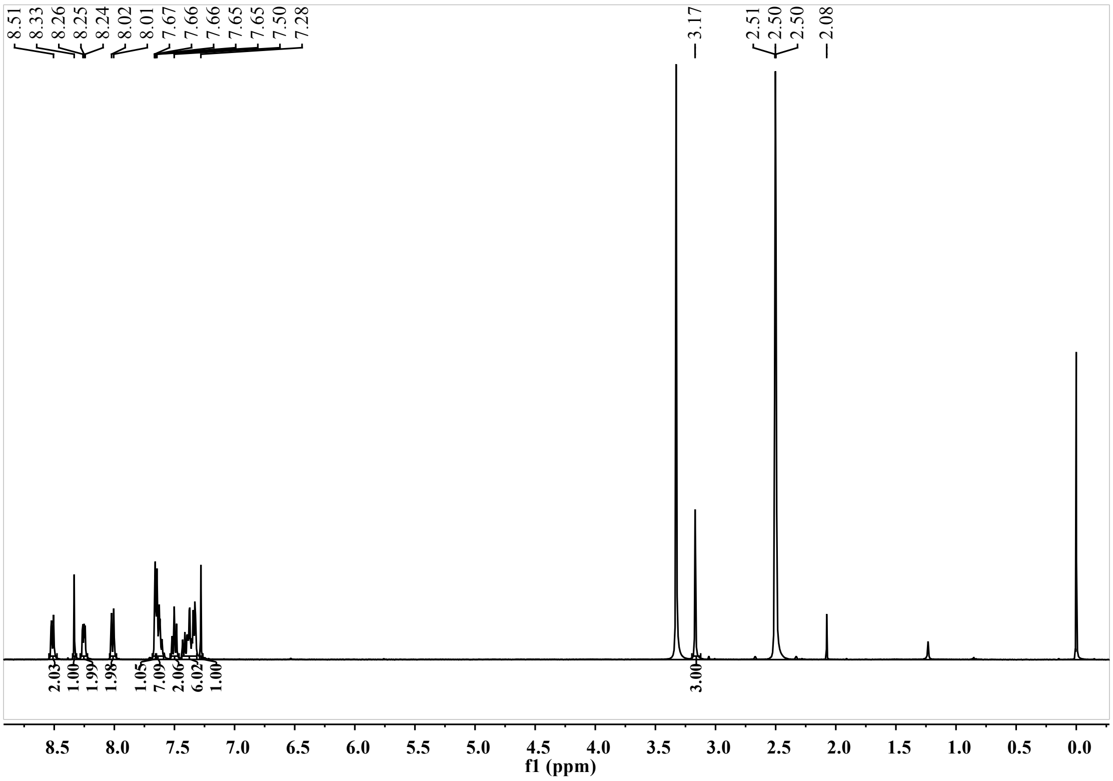

Fig.S1. The^{1}H NMR (400 M, d_6-DMSO) spectra of 2-(3,5-Diphenyl-pyrazol-1-yl)-4-methyl-6,8-diphenyl-1,5,8a,9-tetraaza-fluorene **(L)**

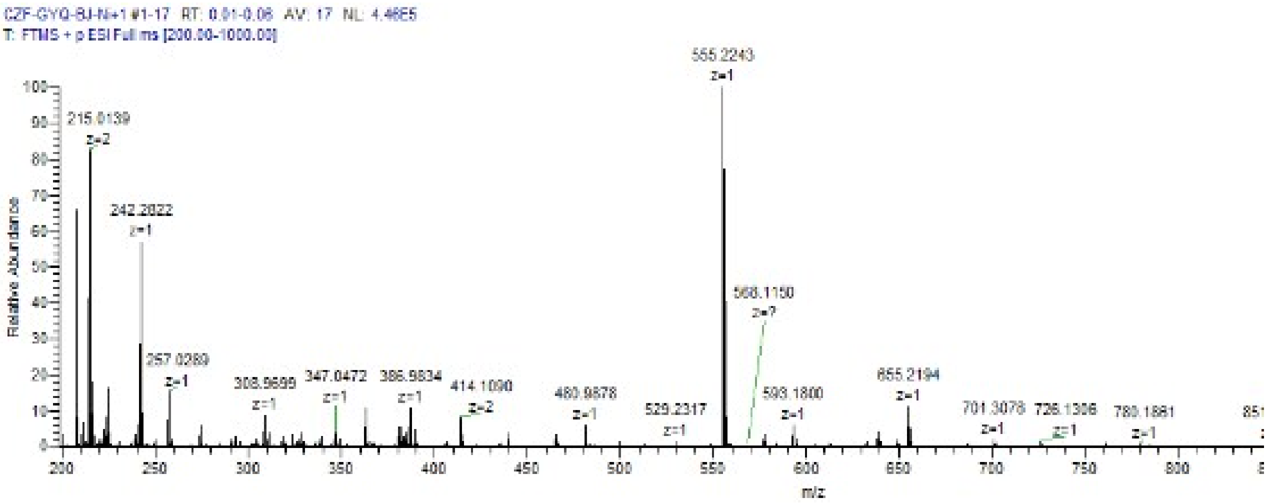

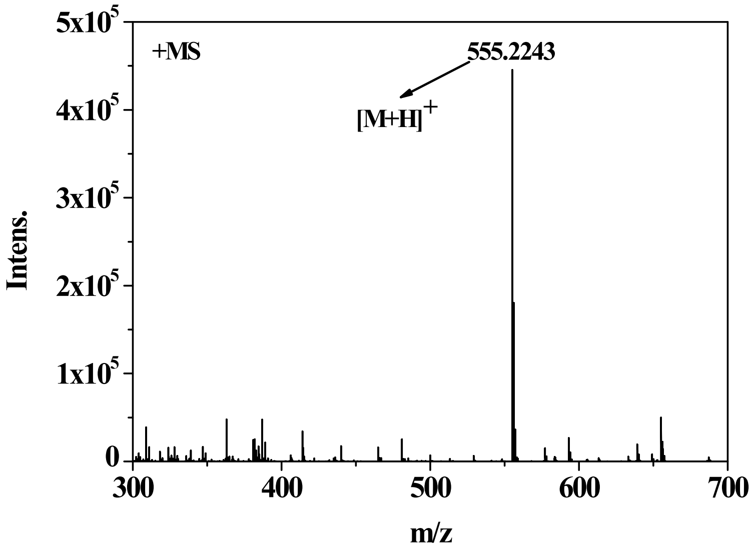

Fig.S2.HRMS spectrum of ligand **L**

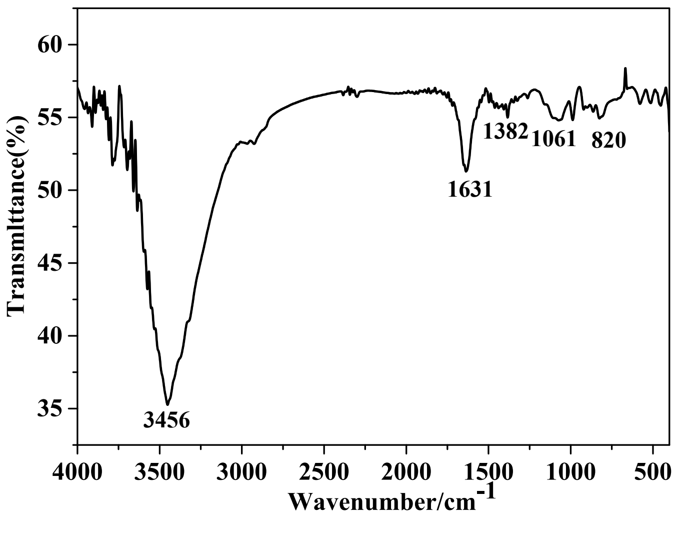

Fig.S3. FT-IR spectra of **L**

Table S1 Crystallographic data and refinements of complexes **1**and**2**

Complex code	**L**
Chemical formula	$C_{37}H_{26}N_6$
Formula Weight	554.64
Temperature(K)	296(2)
Wavelength(Å)	0.71073
Crystal system, space group	*triclinic,P-1*
a/b/c	*a*=11.6231(19)Å<br>*b*=11.723(4)Å<br>*c* = 11.9007(19)Å
α/β/γ	*α*= 98.741(3)°<br>*β* =113.202(2)°<br>*γ* = 100.380(3)°
Volume(Å^3)	1420.8(5)
Z, Caculated density(g.cm^{-3})	28, 1.408
Absorption coefficient (mm^{-1})	0.127
F(000)	616.0
Crystal size (mm^3)	0.310×0.270×0.200
Theta range for data collection	3.644 to 49.998
Limiting indices	–13 ≤ h ≤ 12, –13≤ k ≤ 13,–14≤ l ≤ 13
Reflections collected / unique	7785/4944[R(int) =0.0214]
Completeness to theta = 25.242	98.6%
Refinement method	Full-matrix least-squares on F^2
Data/restraint/parameters	4944/0/389
Goodness-of-fit on F^2	0.977
Final R indices [I>2sigma(I)]	R_1 = 0.0458, wR_2 = 0.1274
R (all data)	R_1 =0.0694, wR_2 = 0.1451
Extinction coefficient	n/a
Largest diff. peak/hole(e/Å^3)	0.514 and -0.352

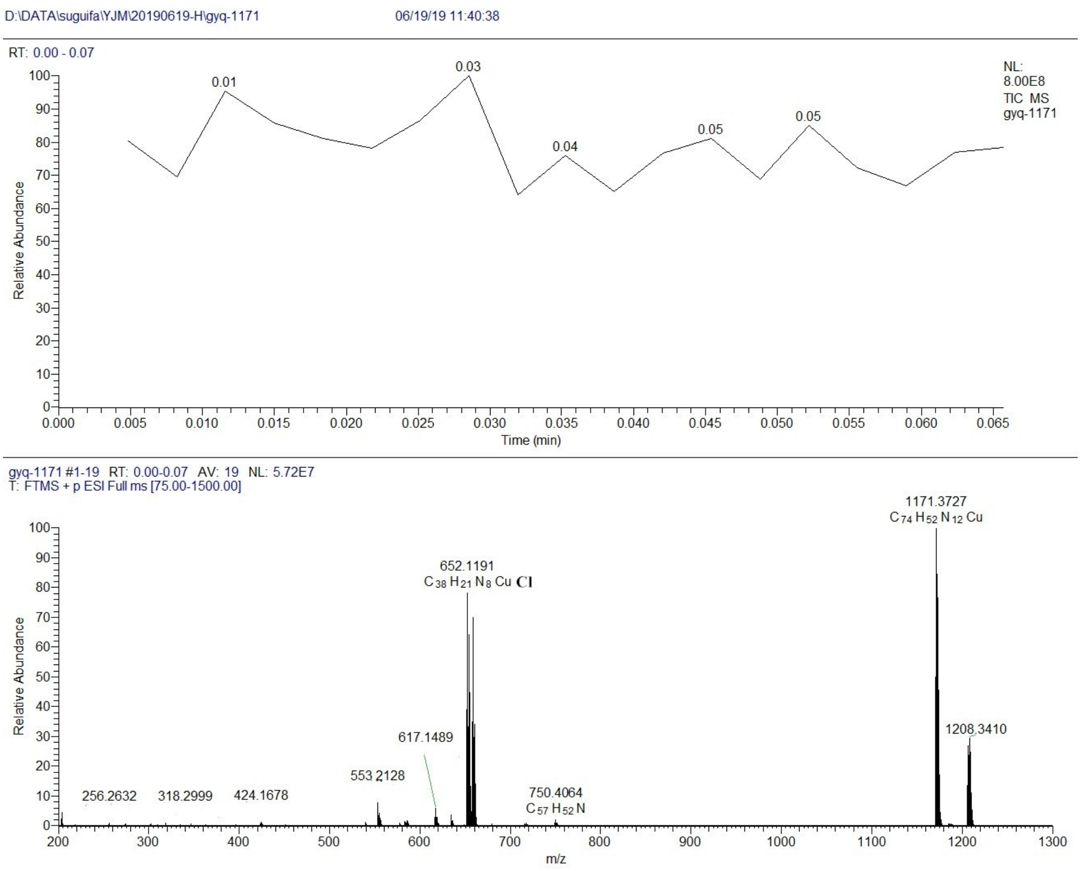

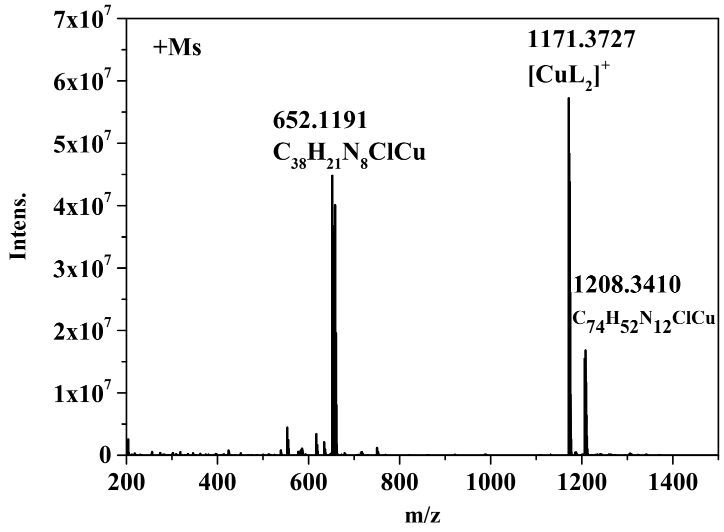

Fig.S4.HRMS spectrum of Cu complex

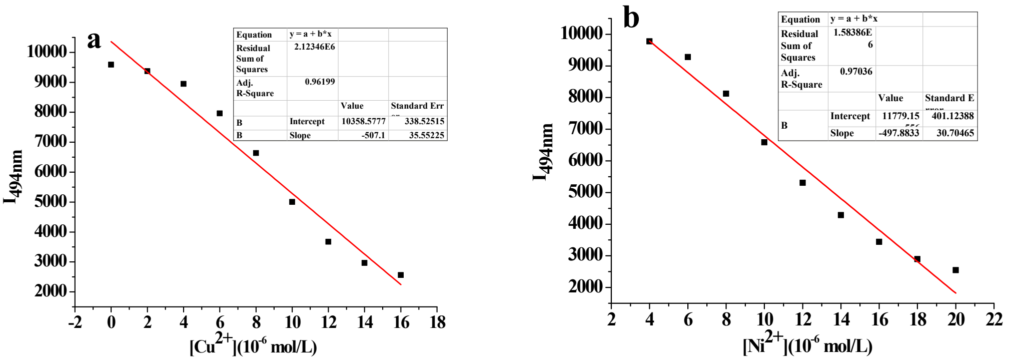

Fig. S5. Calculation of detection limits of **L** for Cu^{2+} (a) and Ni^{2+} (b) in EtOH solution. The spectra were recorded with excitation wavelength at 330 nm and emission wavelength at 494 nm. Fluorescence intensity is the sensor **L** solution (20 μM) upon addition of different amounts of Cu^{2+}/ Ni^{2+}, respectively.

Eq. S1: The detection limit (DL) of Cu^{2+} and Ni^{2+}ions using sensor **L** was determined from the following equation: DL(Cu^{2+}) = $3\sigma/S = 3\times1.47\times10^{-6}/507.1 = 8.7\times10^{-9}$ M, DL(Ni^{2+}) = $3\sigma/S = 3\times1.47\times10^{-6}/497.9 = 8.9\times10^{-9}$ M, respectively, where σ is the standard deviation of the blank solution (10 times); *S* is the slope of the calibration curve.

Table S2. Cytotoxicity of the tested compound **L** against different cell lines (IC_{50} in μM)

T-24	Hela	HepG-2	MGC-803	A549	H-460	Skov-3	HL-7702
39.94±1.35	31.11±0.96	36.14±2.30	30.14±2.42	39.59±1.59	31.73±1.97	38.19±1.68	31.18±2.26
